# Face mask exemptions, psychiatric patients, and COVID-19

**DOI:** 10.1192/j.eurpsy.2020.107

**Published:** 2020-12-07

**Authors:** José Luis Ayuso-Mateos, Joan B. Soriano, Julio Ancochea

**Affiliations:** 1 Department of Psychiatry, Universidad Autónoma de Madrid, Instituto de Investigación Sanitaria Princesa (IIS-Princesa), CIBERSAM, Madrid, Spain; 2 Department of Pneumology, Hospital Universitario de la Princesa, Universidad Autónoma de Madrid, Centro de Investigación en Red de Enfermedades Respiratorias (CIBERES), Instituto de Salud Carlos III (ISCIII), Madrid, Spain

**Keywords:** Anxiety, COVID-19, face masks, psychiatric patients

## Abstract

It has been compulsory to wear face masks in all public spaces, both indoor and outdoor, since May 21, 2020 throughout Spain [1], a measure intended to prevent the spread of coronavirus disease 2019 (COVID-19). However, there are some exceptions to this rule, including “… *People for whom the use of a mask is inadvisable for duly justified health reasons, or who, due to their disability or dependency, present behavioural alterations that make its use unviable.*”

It has been compulsory to wear face masks in all public spaces, both indoor and outdoor, since May 21, 2020 throughout Spain [1], a measure intended to prevent the spread of coronavirus disease 2019 (COVID-19). However, there are some exceptions to this rule, including “… *People for whom the use of a mask is inadvisable for duly justified health reasons, or who, due to their disability or dependency, present behavioural alterations that make its use unviable.*”

Such exemptions are not unique to Spain. For example, in England wearing a face covering is mandated since July 2020, but exemptions include “… *not being able to put on, wear or remove a face covering because of a physical or mental illness or impairment, or disability*” [2].

We believe that the generalized use of this exemption may carry significant risk of COVID-19 for many of the millions of mental patients in Spain, England, or elsewhere worldwide [3]. A recent paper by Wang et al. [4], concludes that patients diagnosed with a mental disorder in the last 12 months have a significantly higher risk of COVID-19 infection than patients without mental disorders, and they also present a worse outcome, as evidenced by higher rates of hospitalization and death.

Although several research groups have already examined the possible future magnifier effects of COVID-19 on mental health, and how to mitigate them [5, 6], there is still very limited evidence regarding how the use of face masks can affect mental health. A recent study from Poland examined the impact of face mask mandates on psychopathological manifestations, and found lower scores on all of the General Health Questionnaire-28 (GHQ-28) subscales after the implementation of such mandates [7]. We agree with these authors’ findings that face masks not only provide protection against COVID-19, but also increase the individuals’ level of perceived self-protection, as well as the level of social solidarity, thereby improving mental wellbeing. A study conducted between March and early April 2020 in healthcare workers across three regions in China found that the strongest significant risk factor for depression was reporting difficulty obtaining face masks [8].

There are medical and legal criteria to orient the debate on whether medical exemptions for masking are necessary or appropriate [9], and indeed similar exemptions for respiratory patients have been strongly questioned [10, 11]. Somehow, the ongoing uncertainty resembles past exemptions on tobacco restriction is psychiatric venues [12]. We concur that most/all health systems will need to adapt the delivery of mental health care to the demands of COVID-19 and both its direct and indirect consequences [13].

For our part, we strongly call on clinicians to be extremely cautious in facilitating any face mask exemption certificates to their patients, given the very limited available evidence on face masks and COVID-19 impacting mental health, and the complete absence of evidence that the potential unpleasantness of wearing a face mask and any associated anxiety might destabilize psychiatric patients. On the contrary, the emphasis should be on trying to educate everyone—regardless of their mental health status—about how to reduce exposure to COVID-19. As to that minority of cases in which, after a personalized assessment of the individual and contextual circumstances, clinicians consider that there are factors *which make the use of a mask unviable,* other measures such as other protective equipment, limiting external contacts or exposures, and else, should be recommended to minimize exposure to COVID-19.
